# MicroRNAs as Prognostic Biomarkers and Therapeutic Targets in Chondrosarcoma

**DOI:** 10.3390/ijms25063176

**Published:** 2024-03-09

**Authors:** Palina Skipar, Mritunjoy Dey, Jakub Piątkowski, Dorota Sulejczak, Piotr Rutkowski, Anna M. Czarnecka

**Affiliations:** 1 Department of Soft Tissue/Bone Sarcoma and Melanoma, Maria Sklodowska-Curie National Cancer Research Institute in Warsaw, 02-781 Warsaw, Poland; palinaskipar@gmail.com (P.S.); mritunjoy.dey@nio.gov.pl (M.D.); piotr.rutkowski@nio.gov.pl (P.R.); 2 Faculty of Medicine, Warsaw Medical University, 02-091 Warsaw, Poland; 3 Institute of Genetics and Biotechnology, Faculty of Biology, University of Warsaw, 02-106 Warsaw, Poland; j.piatkowski@biol.uw.edu.pl; 4 Department of Experimental Pharmacology, Mossakowski Medical Research Centre Polish Academy of Sciences, 02-106 Warsaw, Poland; dsulejczak@imdik.pan.pl

**Keywords:** chondrosarcoma, microRNA, primary malignant bone tumor

## Abstract

Chondrosarcoma, the second most common primary malignant bone tumor, originates from cartilaginous tissue and accounts for almost 20% of all primary bone tumors. The management of chondrosarcoma remains challenging due to its diverse clinical course and prognosis, which can range from benign to highly aggressive with a huge risk of metastasis. Emerging research has demonstrated the importance of microRNA (miRNA) dysregulation in the pathogenesis of chondrosarcoma. MiRNAs are small, noncoding RNA molecules that play an essential role in gene expression regulation by targeting specific messenger RNAs (mRNAs) for degradation or translational repression. This article provides an extensive review of current miRNA research in chondrosarcoma, focusing on diagnostic strategies, cell cycle regulation, drug resistance, biomarkers of progression, and stem cell phenotype. We will examine recent studies identifying differentially expressed miRNAs in chondrosarcoma compared to normal cartilage tissue, exploring their potential as diagnostic and prognostic biomarkers. Furthermore, we will discuss the role of miRNAs in regulating cell cycle progression and their potential as therapeutic targets to overcome drug resistance. We will also investigate the prospective utility of miRNAs as biomarkers of progression and their role in modulating the stem cell phenotype of chondrosarcoma cells. This article offers a comprehensive analysis of current miRNA research in chondrosarcoma, focusing on its potential as diagnostic and prognostic biomarkers, therapeutic targets, and regulators of disease progression. By integrating the latest discoveries in this field, we aim to contribute to the development of novel approaches to the prevention, diagnosis, and treatment of chondrosarcoma, ultimately enhancing patient outcomes.

## 1. Introduction

Chondrosarcoma is a primary malignant bone tumor that originates in cartilaginous tissue. It represents approximately 20% of all bone tumors and is the second most common malignant bone tumor after osteosarcoma. Despite advances in treatment, chondrosarcoma remains challenging to manage due to its highly variable clinical course and prognosis, ranging from benign to highly aggressive behavior with a high risk of metastasis.

Recent studies have shown that dysregulation of microRNA (miRNA) expression is involved in the pathogenesis of chondrosarcoma. MiRNAs are short, noncoding endogenous transcripts that participate in all manners of crucial cellular processes. They act mainly by means of translational repression and induce mRNA degradation [1,2], but under specific conditions, they have also been found to activate translation [3,4,5,6]. MiRNA genes are found within introns of protein-coding genes (far more rarely within exons) or as separate genes with independent promoters [7,8]. These genes are transcribed into primary miRNAs (pri-miRNAs) by RNA polymerase II/III and then processed into pre-miRNAs and finally into mature miRNAs. At the time of writing of this article, miRBase contained 4496 sequences of human miRNAs, including 1864 pre-miRNAs and 2631 mature miRNAs [9]. The majority of human coding transcripts contain predicted miRNA target sites [10]. The said target sites are located primarily, but not exclusively, within 3’UTRs. Aberrations in miRNA expression have been linked to multiple human diseases, including cancer. MiRNAs can act as both oncogenes and tumor suppressors. Different types of cancer display a high level of diversity in terms of miRNA expression, making miRNA expression profiles potentially very informative for classifying human cancers. However, global down-regulation of miRNAs is observed in different types of cancer [11].

In this paper, our objective is to provide a comprehensive summary of current research on miRNAs in chondrosarcoma, with a focus on several key areas, including diagnostic approaches, cell cycle regulation, drug resistance, biomarkers of progression, and stem cell phenotype. By examining the latest research in these areas, we hope to contribute to the ongoing effort to develop more effective approaches to chondrosarcoma prevention, diagnosis, and treatment. In particular, we will review recent studies that have identified differentially expressed miRNAs in chondrosarcoma compared to normal cartilage tissue and their potential as diagnostic and prognostic biomarkers. We will also discuss the role of miRNAs in regulating cell cycle progression and their potential as therapeutic targets for overcoming drug resistance. In addition, we will examine the potential of miRNAs as biomarkers of progression and their role in regulating the stem cell phenotype of chondrosarcoma cells.

In general, this article will provide a comprehensive overview of current research on miRNAs in chondrosarcoma, highlighting their potential as diagnostic and prognostic biomarkers, therapeutic targets, and regulators of disease progression. By synthesizing the latest findings in this field, we hope to contribute to the development of new approaches to the prevention, diagnosis, and treatment of chondrosarcoma, and ultimately improve patient outcomes.

## 2. Diagnostics and Biomarkers of Progression

**Figure 1:**
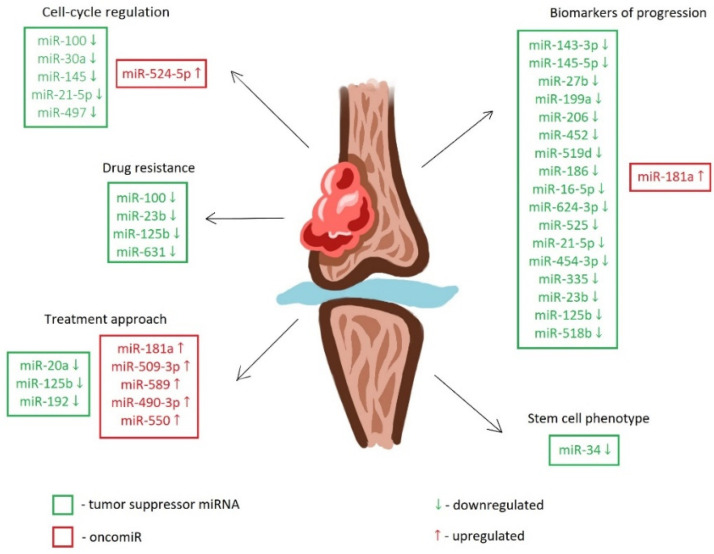
MiRNAs in chondrosarcoma. Down-regulated tumor suppressor miRNAs and up-regulated oncomiRs affect cell cycle regulation, drug resistance, progression, stem cell phenotype, and treatment targets.

MiRNAs show great potential as biomarkers for the early detection and prognosis of chondrosarcoma. Their stability in biological fluids and tissue-specific expression patterns make them ideal candidates for non-invasive liquid biopsies [12]. In chondrosarcoma, miRNA dysregulation has been found to impact various aspects of tumor progression, including cell proliferation, apoptosis, invasion, metastasis, and angiogenesis. Oncogenic miRNAs, or oncomiRs, typically promote tumor progression by down-regulating the expression of tumor suppressor genes or improving oncogene activation. On the contrary, tumor-suppressive miRNAs inhibit the development of chondrosarcoma by targeting and down-regulating oncogenes. The table below illustrates the target genes and miRNA expression in chondrosarcoma (Table 1, Figure 1).Figure 1 depicts differentially regulated microRNAs in chondrosarcoma.

Recent studies have identified various miRNAs that play a crucial role in chondrosarcoma progression by regulating tumor metastasis, angiogenesis, and lymphangiogenesis. These miRNAs show promise as potential biomarkers to monitor the progression of chondrosarcoma and as therapeutic targets. Fascin-1 (FSCN1), an actin-bundling protein, organizes the arrangement of actin filaments into parallel bundles, thus participating in a range of physiological cellular processes including the regulation of cell adhesion, motility, migration, and interactions. FSCN1 has been observed to be a direct target of several tumor suppressor miRNAs, such as miR-143-3p and miR-145-5p. A study [13] demonstrated that down-regulation of these miRNAs in chondrosarcoma triggers up-regulation of FSCN1, thus promoting tumor progression and metastasis. Thus, this highlights the crucial role of FSCN1 and the miRNAs that regulate it in the pathogenesis of chondrosarcoma.

F-spondin 1 (SPON1), an extracellular matrix protein known for enhancing neuronal development, emerges as a potential player in chondrosarcoma. While studies demonstrate its ability to promote neuronal attachment and growth, and even activate pro-tumorigenic pathways in osteosarcoma, its specific role in chondrosarcoma remains unclear. However, a compelling clue lies in the significantly lower levels of miR-525, a microRNA directly targeting SPON1, observed in chondrosarcoma patients. This suggests a potential regulatory pathway: decreased miR-525 expression could contribute to increased malignancy by deregulating SPON1, leading to enhanced cell migration and invasion [45]. This indicates miR-525′s potential as a biomarker for the progression of chondrosarcoma.

STAT3, a central regulator of tumor cell metabolism, emerges as a potential target in chondrosarcoma due to its involvement in multiple pro-tumorigenic pathways. Decreased miR-21-5p levels fuel chondrosarcoma progression by activating the CCR7/STAT3/NF-κB axis, promoting proliferation, migration, and invasion. Notably, miR-21-5p directly targets CCR7, suppressing downstream STAT3 and NF-κB signaling—crucial players in cancer development [34]. Similar to miR-21-5p, miR-454-3p, another down-regulated miRNA in chondrosarcoma tissues, exerts a regulatory effect on STAT3. Interestingly, increased expression of the long non-coding RNA HOTAIR leads to miR-454-3p up-regulation, subsequently suppressing STAT3 and ATG12, triggering apoptosis, decreasing autophagy, and inhibiting chondrosarcoma cell growth [49]. This convergent targeting of STAT3 across different pathways highlights its potential as a focal point for developing novel, targeted therapies against chondrosarcoma.

MiR-335, consistently down-regulated in chondrosarcoma [21], emerges as a potential player in tumor progression and metastasis. This aligns with observations in breast cancer, where decreased miR-335 expression correlates with poor survival due to its inhibitory effect on metastasis via targeting SOX4 and Tenascin-C (TNC) [50]. While this study focused on breast cancer, the similar down-regulation in chondrosarcoma suggests a potential parallel mechanism, implicating TNC in chondrosarcoma progression. This notion is further supported by the crucial role of TNC, highly expressed in the tumor stroma, in promoting chondrosarcoma cell survival via TNC-mediated adhesion and Akt activation. This convergence highlights both miR-335 and TNC as potential nodes for therapeutic intervention in chondrosarcoma.

The observed down-regulation of miR-23b has been correlated with an increase in the activity of Src kinase [51]. Src kinase, a vital factor in several subtypes of sarcomas, including chondrosarcoma, exerts its tumorigenic influence through the enhancement of cell proliferation, the reduction of apoptosis, and the promotion of metastasis. Therefore, increased Src activity caused by decreased miR-23b expression has implications for more aggressive tumor behavior. Simultaneously, a reduction in miR-125b levels promotes the proliferation of chondrosarcoma cells, exerting this effect through its targeted regulation of ErbB2 [52]. This miRNA–tumor protein interaction is critical, as ErbB2, when not controlled, is associated with enhanced cell motility and invasiveness. The inhibitory function of miR-125b in ErbB2 not only impacts glucose metabolism but also increases the sensitivity of chondrosarcoma cells to the chemotherapeutic drug doxorubicin. Therefore, a decrease in miR-125b levels could result in increased resistance to chemotherapy, marking it as a potential target for therapeutic interventions.

Rap1b, a member of the Ras superfamily of small GTPases, has established roles in cellular processes such as angiogenesis and cell migration, both of which are critical in cancer progression. In the context of esophageal squamous cell carcinoma (ESCC), miR-518b, which acts as a tumor suppressor, induces apoptosis and represses invasion by specifically targeting Rap1b [46]. Recent findings have identified that gallic acid increases miR-518b expression in human chondrosarcoma cells, leading to the promotion of apoptosis and the inhibition of cell migration [47]. Given these observations, it is plausible that miR-518b may exert a tumor suppressor effect similar to that observed in chondrosarcoma as observed in ESCC, potentially by targeting Rap1b. This observation, while full of potential, requires further research. We need to better understand how miR-518b works with Rap1b, and what role this relationship could play in the development and progression of chondrosarcoma.

Angiogenesis, the formation of new blood vessels from preexisting vasculature, is an essential process in the pathophysiology of cancer, including chondrosarcoma. This process allows fast-growing tumor cells to receive necessary nutrients and oxygen, supporting their survival, growth, and potential spread. The molecular intricacies that underlie angiogenesis primarily involve vascular endothelial growth factor (VEGF) and its derivatives, VEGF-A and VEGF-C, which are central to both angiogenesis and lymphangiogenesis. In chondrosarcoma, the oncogenic miRNA, miR-181a, is up-regulated under hypoxic conditions, increasing VEGF expression by targeting the G-protein signaling 16 regulator (RGS16). Furthermore, miR-181a negatively modulates the CXC chemokine receptor 4 (CXCR4) signaling pathway, further affecting tumor progression [16]. In particular, CXCR4 contributes to the invasion of chondrosarcoma by up-regulating multiple genes, including the alphavbeta3 integrin and various matrix metalloproteinases (MMPs).

VEGF-A, a variant of VEGF, significantly contributes to angiogenesis. Its expression is induced by the suppression of miR-27b [36] and down-regulation of miR-199a through CCL5. Furthermore, CCL5 stimulates cell migration through the activation of matrix metalloproteinase-3 (MMP-3), thus aiding in the metastasis of chondrosarcoma [27]. Increased expression of VEGF-A also results from inhibition of miR-206 by amphiregulin [28] and miR-452 by Wnt-induced secreted protein-3 (WISP-3) [29]. Importantly, resistin, an adipokine, promotes VEGF-A-driven angiogenesis by suppressing miR-16-5p [53], while also promoting chondrosarcoma metastasis by down-regulating miR-519d and enhancing MMP-2 expression through the AMPK/p38 signaling pathway [54].

VEGF-C, another form of VEGF, regulates the creation of lymphatic vessels, thereby facilitating tumor spread through the lymphatic system. The suppression of miR-27b triggers the expression of VEGF-C [36]. Leptin, an adipocyte-derived hormone, accelerates VEGF-C production and promotes lymphangiogenesis by suppressing miR-27b [37]. Furthermore, resistin aids VEGF-C-associated lymphangiogenesis by suppressing miR-186 [35], while brain-derived neurotrophic factor (BDNF) improves VEGF-C-dependent lymphangiogenesis by suppressing miR-624-3p [38].

These findings provide insight into the roles of miRNAs in chondrosarcoma progression and their potential as prognostic biomarkers and therapeutic targets for this malignancy.

## 3. MicroRNAs as Regulators of the Cell Cycle

Understanding the molecular mechanisms that underlie the development and progression of chondrosarcoma is crucial for the identification of new therapeutic targets. In recent years, miRNAs have emerged as key players in this context. These small noncoding RNAs modulate gene expression and have been found to influence a variety of cellular processes in chondrosarcoma, including regulation of the cell cycle.

An example involves miR-100, a tumor suppressor miRNA that plays a significant role in chondrosarcoma progression by targeting and inhibiting the mammalian target of the rapamycin (mTOR) signaling pathway, which is involved in tumor growth and metastasis. Decreased miR-100 levels lead to the activation of mTOR signaling, resulting in increased cell proliferation and invasion of healthy tissues. Therefore, restoring miR-100 expression could be a potential therapeutic approach to prevent chondrosarcoma progression by suppressing aberrant activation of the mTOR pathway [55]. Another miRNA, miR-30a, exhibits the ability to decrease tumor proliferation, migration, and invasion in chondrosarcoma by targeting the oncogenic SRY-related HMG box 4 (SOX4), which is involved in chondrocyte differentiation [30]. Down-regulation of miR-30a leads to increased expression of SOX4, which cooperates with various transcription factors for the genesis of chondrosarcomas, such as c-MYC, E2F1, and E2F4, suggesting tumor progression. MiR-30a is also known to negatively target RUNX2 in CS cells, inhibiting this gene to enhance cancer invasion [31]. RUNX2 (Runt-related transcription factor 2) is a transcription factor that plays a critical role in skeletal development and bone formation. In the context of chondrosarcoma, RUNX2 may promote cancer progression by regulating genes involved in cell proliferation, invasion, and angiogenesis. These studies suggest that miR-30a is an important tumor suppressor. Low miR-145 expression corresponds to elevated levels of another SOX gene, SOX9, a significant transcription factor involved in the specification, differentiation, and survival of the chondrocyte lineage. Overexpression of miR-145 reduces SOX9 expression, inhibiting proliferation and invasion of chondrosarcoma cells [56]. Furthermore, decreased miR-21-5p levels contribute to chondrosarcoma cell proliferation, migration, and invasion through activation of the CCR7/STAT3/NF-κB pathway. MiR-21-5p directly targets CCR7, coordinating cancer cell migration, inhibiting its expression, and, therefore, suppressing downstream STAT3, which regulates tumor cell metabolism and NF-κB signaling, mediating neoplasm proliferation, survival, and angiogenesis [34]. In human chondrosarcoma samples and cells, miR-497 is down-regulated. Its overexpression reduces proliferation and enhances apoptosis in chondrosarcoma cells by targeting Cdc25A, a regulator of apoptosis, through a p53-independent pathway. This suggests that miR-497 could serve as a potential tumor suppressor and therapeutic target for chondrosarcoma [42]. Furthermore, the YAP/miR-524-5p axis regulates the tumor suppressor gene TXNIP (Thioredoxin-Interacting Protein) in chondrosarcoma, inhibiting tumor cell proliferation and promoting tumor cell apoptosis by participating in metabolic reprogramming, including the control of glucose utilization and oxidative stress. Targeting this axis might offer a promising therapeutic strategy for the management of chondrosarcoma [57].

MiRNAs appear to be a promising pathway for anticancer therapy. Veys et al. investigated the antiproliferative and chemo-enhancing potential of selected miRNAs in SW1353 chondrosarcoma cells. They examined the chemotherapy sensitization capacity and antiproliferative potential of selected miRNAs in chondrosarcoma cells. Studies conducted on three chondrosarcoma cell lines showed that miR-342-5p has a strong ability to suppress chondrosarcoma by affecting the expression of Bcl-2 and Bcl-xL proteins and inducing apoptosis or autophagy. In contrast, miR-491-5p showed weaker tumor suppressive effects and inhibited the expression of Bcl-xL and EGFR [41].

An interesting study by Rémy et al. showed that the loss of a microRNA cluster at the 14q32 locus is involved in the progression of chondrosarcoma. The authors observed a decrease in 14q32-located microRNA expression and a decrease in the levels of other microRNAs such as miR-27B, miR-125A, and miR-140, and found them to be important determinants of the disease process that could be helpful in [58].

The impact of miRNAs on cell cycle regulation in chondrosarcoma sheds light on the complex molecular mechanisms driving this aggressive bone cancer. Studying the role of miRNAs in cell cycle progression and other essential cellular processes can lead to identifying potential therapeutic targets and innovative management strategies for chondrosarcoma. A deeper understanding of miRNA function in the pathogenesis of chondrosarcoma may ultimately result in more effective treatment options and improved patient outcomes.

## 4. MicroRNAs as Regulators of Drug Resistance

Chondrosarcoma often exhibits resistance to conventional chemotherapy, leading to poor treatment outcomes. The development of drug resistance is a major obstacle in the management of chondrosarcoma. Recent studies have highlighted the role of miRNAs in modulating drug resistance, suggesting their potential as therapeutic targets to address this issue. This section discusses the impact of miRNAs on drug resistance in chondrosarcoma and explores potential therapeutic strategies based on these findings.

The overexpression of miR-100 and miR-23b has been found to sensitize cisplatin-resistant chondrosarcoma cells, making them more receptive to cisplatin treatment. This discovery highlights the potential therapeutic value of miR-100 and miR-23b as an adjunct to chemotherapy in chondrosarcoma treatment. Enhancing their expression could potentially overcome resistance to cisplatin and improve the efficacy of chemotherapy in patients with chondrosarcoma [51,55]. Similarly, low levels of miR-125b have been associated with resistance to doxorubicin in chondrosarcoma cells in vitro. Enhancing miR-125b expression could be a viable strategy to overcome resistance to doxorubicin in this malignancy [52]. Furthermore, miR-631 has been found to restore sensitivity in doxorubicin-resistant chondrosarcoma cells by targeting apelin, a peptide involved in the promotion of angiogenesis, metastasis, cell proliferation, and the development of cancer stem cells and drug resistance [48]. This finding presents another promising avenue for improving chondrosarcoma treatment by addressing drug resistance.

Recent advancements in the study of miRNAs have opened new avenues in combating drug resistance in chondrosarcoma. A pivotal study by Vares et al. (2020) proposes an innovative approach using rapamycin to inhibit the mTOR pathway, along with a miR-34 mimic, as a strategy to break down the radioresistance commonly observed in cancer stem cells within chondrosarcoma during carbon-ion therapy [44]. This combination therapy could not only improve the success rate of carbon-ion treatment at reduced doses but also potentially reduce the risk of cancer recurrence and metastasis while protecting nearby healthy tissue from damage.

Complementary research, such as that by Tuddenham et al. (2006), although not directly focused on chondrosarcoma, offers valuable insights for future studies. MiR-140, a chondrocyte-specific miRNA, has been identified as crucial in the growth and invasion of chondrosarcoma cells by reducing HDAC4 expression. Intriguingly, this miRNA also enhances the sensitivity of breast cancer cells to doxorubicin by modulating PD-L1 levels, suggesting its broader role in mediating drug response in various cancers [59,60].

Another significant microRNA is miR-34a, which seems to play an indirect, yet critical role in the treatment of drug resistance in chondrosarcoma. This miRNA primarily affects cellular processes such as apoptosis and senescence [61]. Research, including a 2020 study on multimodal treatment for high-grade chondrosarcoma, underscores the role of miR-34a in modulating cancer stem cell behavior, a key factor in overcoming therapeutic resistance [44]. Given that cancer stem cells play a pivotal role in evading standard treatments, targeting them with miR-34a could indirectly help resensitize chondrosarcoma cells to various therapies, thus potentially increasing the overall effectiveness of concurrent treatment modalities [62].

MiR-199 has been found to reverse cisplatin resistance in human ovarian cancer cells by inhibiting mTOR, a pathway that could also be a target of miR-199a in chondrosarcoma [63]. This connection is further reinforced by the discovery that miR-199 is encoded by Dnm3os, a long RNA transcript. Previous research has shown that the loss of Dnm3os leads to defects in endochondral bone growth in mice, hinting at its relevance in bone-related cancers such as chondrosarcoma [64].

Further studies show that miR-424 regulates the PD-L1/PD-1 and CD80/CTLA-4 pathways in drug-resistant ovarian cancer [65], and restoration of its expression reverses the chemoresistance that accompanies PD-L1 immune checkpoint blockage [66].

Recent understanding of the role of miR-27a in lung adenocarcinoma shows that it is up-regulated in cisplatin-resistant cells compared with sensitive cells. MiR-27a has been identified to regulate the epithelial–mesenchymal transition (EMT) and cisplatin resistance process, primarily by targeting the Raf Kinase Inhibitory Protein (RKIP). The suppression of RKIP expression by up-regulated miR-27a contributes significantly to chemoresistance [67]. This is relevant since miR-27 is enriched in the pharyngeal arches in zebrafish, a key area for cartilage development. In particular, pharyngeal cartilage was lost when miR-27 was removed, implicating its role in chondrogenesis through the regulation of Ptk2aa, a focal adhesion kinase, and a negative regulator of chondrogenesis [68].

In colon cancer, miR-195 is significantly down-regulated in cells resistant to doxorubicin, and its reduction further improves this resistance, indicating its role in sensitizing cells to the drug by targeting the expression of BCL2L2 [69]. Significantly, in the context of chondrosarcoma, miR-195 targets Git1 in chondrocyte cells, suppressing their proliferation and migration [70,71].

In breast cancer, miR-222 overexpression in doxorubicin-resistant cells facilitates increased proliferation and migration while reducing apoptosis via the miR-222-Bim-caspase pathway [72]. Similarly, in bladder cancer, miR-222 overexpression contributes to resistance to cisplatin by activating the Akt/mTOR pathway and inhibiting autophagy [73]. These observations suggest a potential parallel in chondrosarcoma since miR-222 is inhibited in human mesenchymal stem cells, which promotes chondrocyte differentiation. Furthermore, in rat models, local inhibition of miR-222 enhances chondrogenesis and osteogenesis, indicating its potential role in the biology of chondrosarcoma and responsiveness to therapy, although direct evidence in chondrosarcoma remains to be established [74].

MiR-146a contributes to resistance to doxorubicin in breast cancer by altering cell signaling and apoptosis and similarly influences resistance to cisplatin in non-small cell lung cancer (NSCLC) through the NF-κB pathway. In particular, lower levels of miR-146a are associated with increased resistance to cisplatin in NSCLC [75,76]. In chondrosarcoma, its variant, miR-146b, affects chondrocyte differentiation by modulating Sox5 and Sox6, key chondrogenesis regulators. This illustrates the complex role of microRNA-146a and its variants in various types of cancer, highlighting its potential to understand drug resistance and tissue differentiation [77,78].

These potentially pivotal connections remain to be fully explored in chondrosarcoma. Emerging patterns suggest that miRNAs, such as miR-34a, miR-199, and miR-222, may play crucial roles in modulating drug resistance, not just in chondrosarcoma but across a spectrum of cancers. These connections, particularly in the regulation of cancer stem cells, apoptosis, and cellular differentiation, open promising avenues for innovative therapies. The multifaceted nature of miRNA interactions in chondrosarcoma highlights the need for further research to unravel these complex biological networks, which could lead to more effective strategies to combat drug resistance in chondrosarcoma and other cancers.

## 5. MiRNA-Based Treatment Approaches in Chondrosarcoma

MicroRNAs (miRNAs) hold promise as a novel therapeutic approach for chondrosarcoma. Researchers are exploring diverse strategies, including silencing oncogenic miRNAs with anti-miRNA oligonucleotides, inducing tumor suppressor miRNAs with drugs like PRP-1, and targeting stem cell characteristics with miR-34. Additionally, miRNA sponges and lncRNAs, while not yet tested in chondrosarcoma specifically, offer potential avenues for future exploration. These approaches offer hope for developing new and effective treatments for this challenging cancer type.

Sun et al. devised a method of utilizing anti-miRNA oligonucleotides (AMOs) directed against miR-181a utilizing a nanopiece delivery platform (NP). The study employed a novel approach utilizing nanopiece delivery platforms (NPs) loaded with anti-miR-181a oligonucleotides for systemic targeting of miR-181a. Both intratumoral and systemic delivery of NP-conjugated anti-miR-181a efficiently reduced miR-181a expression in xenograft models, consequently inhibiting the expression of downstream genes like RGS16 and CXCR4 and ultimately leading to tumor growth suppression. The nanoparticle delivery system enhances the specificity and efficacy of AMO delivery, potentially minimizing undesirable side effects and improving therapeutic outcomes [17].

Another promising strategy employs cytostatic antiproliferative proline-rich polypeptide (PRP-1) [79]. This molecule inhibits the mammalian target of rapamycin complex 1 (mTORC1), a central regulator of protein synthesis. Treatment with PRP-1 in human chondrosarcoma cell lines has been shown to up-regulate tumor suppressor miRNAs, such as miR-20a, miR-125b, and miR-192, while simultaneously down-regulating oncogenic miRNAs like miR-509-3p, miR-589, miR-490-3p, and miR-550. These findings suggest that PRP-1, by modulating miRNA expression, could have significant therapeutic potential in controlling tumor progression within chondrosarcoma.

MicroRNAs also influence the stem cell phenotype of chondrosarcoma cells. This phenotype contributes significantly to both tumor progression and resistance to therapy. Notably, miR-34, a microRNA known for its tumor-suppressive properties, plays a crucial role in modulating these stem-like characteristics. MiR-34 specifically targets genes like NOTCH1, C-MYC, LMTK3, and KLF4, which are involved in various cellular processes related to stem cell function. Studies have demonstrated that miR-34 can suppress stem-like properties in various cancers, and evidence suggests it has a similar effect in chondrosarcoma. MiR-34 expression is typically down-regulated in chondrosarcoma cell lines. Conversely, overexpression of miR-34 has been shown to significantly reduce the invasive potential of these cells and impair their ability to form spheroids, structures associated with stemness, in vitro [43].

Furthermore, there are techniques that have not been tested in chondrosarcoma specifically but have proven their potential in other cancer types. One such technique includes the microRNA “sponge”. MiRNA sponges, introduced in 2007, are synthetic molecules that mimic natural competing endogenous RNAs (ceRNAs) by containing multiple binding sites for specific miRNAs. These sponges act as “sinks” for miRNAs, preventing them from interacting with their natural targets and regulating gene expression [80]. Bioinformatic analysis suggests that circular RNAs (circRNAs), like circ_0078710, circ_0067934, and circ_0103809, may contribute to carcinogenesis. Circ_0078710 exhibits competitive binding with HDAC2 and CDK2, thereby potentially affecting the suppressive function of miR-31 towards oncogenes. Similarly, circ_0067934 and circ_0103809 potentially regulate the miR-1324/FZD5/Wnt/β-catenin and miR-490-5p/SOX2 signaling pathways, respectively, potentially promoting hepatocellular carcinoma (HCC) growth and metastasis [81,82].

The long non-coding RNA (lncRNA) named CRNDE, positioned at chromosome 16q12.2, was first discovered in colorectal cancer [83]. Studies revealed that CRNDE can act as a molecular sponge for miR-136 in breast cancer, consequently enhancing the Wnt/β-catenin signaling pathway and promoting tumor growth [84]. Furthermore, up-regulated CRNDE was observed to function as a sponge for miR-384, contributing to hepatocellular carcinoma (HCC) tumorigenesis. Another example involves the lncRNA XIST, which interacts with the oncogenic miR-181a, often up-regulated in HCC tissues. Research suggests that miR-181a targets PTEN, a tumor suppressor that inhibits the PI3K/Akt pathway. Consequently, down-regulation of XIST may lead to increased miR-181a activity, reduced PTEN levels, and subsequent activation of the pro-tumorigenic PI3K/Akt pathway in HCC [85,86].

Similarly, by functioning as a molecular sponge for miR-106a-5p to control PTEN expression, lncRNA 657 inhibits the development of HCC cells [87]. XIST may target miR-92b to prevent HCC cell growth and metastasis. XIST and miR-92b may also directly interact and repress one another. Zhang et al. discovered that the XIST/miR-92b/Smad7 signaling axis in HCC progression miRNA-92b targets Smad7 to enhance the progression of hepatocellular carcinoma, and this is mediated by the long non-coding RNA XIST [88].

The miR-17-92 cluster, which is often up-regulated in rapidly proliferating cells, has been found to have binding sites particularly enriched just upstream of APA sites. This suggests that the shortening of 3′UTRs might not only enable the escape from inhibition of growth-promoting genes but also potentiate the repression of anti-proliferative genes. In the context of chondrosarcoma, this could potentially have significant implications. If the miR-17-92 cluster is up-regulated in chondrosarcoma cells, the modulation of 3′UTRs by mRNAs could influence the degree of regulation by this miRNA cluster. This could potentially affect the proliferation of the cancer cells and the progression of the disease [89,90]. However, the specific role and mechanisms of the miR-17-92 cluster and 3′UTR modulation in chondrosarcoma would require further research for a comprehensive understanding. It is also important to note that the role of miRNAs and their interaction with mRNAs can vary widely depending on the specific biological context. While established defense mechanisms like 3’UTR shortening, sequence alterations, and competing endogenous RNAs (ceRNAs) exist, their specific role in chondrosarcoma requires further investigation. Current research primarily focuses on how miRNAs regulate target mRNAs, contributing to cancer progression or suppression. However, future studies should investigate deeper into the potential defensive strategies employed by mRNAs in chondrosarcoma, offering a more comprehensive understanding of the complex interplay between these molecules.

This research highlights various promising techniques, including utilizing anti-miRNA oligonucleotides to silence oncogenic miRNAs, employing proline-rich polypeptides to induce tumor suppressor miRNAs, and targeting the stem cell characteristics of cancer cells using miR-34. Additionally, the potential of miRNA sponges and long non-coding RNAs, though not yet explored in chondrosarcoma specifically, warrants further investigation. It is crucial to remember that these approaches are still in their early stages, and further research and clinical trials are necessary to fully establish their efficacy and safety in treating this complex cancer.

## 6. Conclusions

In conclusion, this paper provides a comprehensive analysis of current research on microRNAs (miRNAs) in chondrosarcoma, shedding light on their potential implications for the diagnosis, treatment, and prognosis of this challenging bone tumor. Chondrosarcoma, which is the second most common primary malignant bone tumor, poses significant difficulties in its management due to its variable clinical course and unpredictable outcomes.

The emerging field of miRNA research has demonstrated the crucial role of these small RNA molecules in the pathogenesis of chondrosarcoma. By regulating gene expression through the targeting of specific mRNAs, miRNAs have the ability to influence various aspects of tumor behavior.

This paper highlights the significance of miRNAs as diagnostic and prognostic biomarkers in chondrosarcoma. Through careful examination of differentially expressed miRNAs in chondrosarcoma compared to normal cartilage tissue, researchers have identified promising candidates for early detection and accurate prediction of disease progression. These miRNAs have the potential to improve clinical decision-making and optimize patient care.

Furthermore, this article explores the role of miRNAs in the regulation of the cell cycle and the overcoming of drug resistance in chondrosarcoma. Understanding the mechanisms by which miRNAs influence cell cycle progression and modulate response to treatment is of utmost importance in developing effective therapeutic strategies. Targeting specific miRNAs implicated in drug resistance may offer new avenues to improve treatment outcomes.

Investigating miRNAs as biomarkers of disease progression and their participation in modulating the stem cell phenotype of chondrosarcoma cells provides valuable information on the underlying mechanisms driving tumor development and aggressiveness. These findings have the potential to shape future research directions and guide the development of targeted therapies.

In summary, this paper underscores the significant role of miRNAs in chondrosarcoma, highlighting their potential as diagnostic tools, therapeutic targets, and regulators of disease progression. By advancing our understanding of miRNA dysregulation in chondrosarcoma, this research contributes to ongoing efforts to improve prevention, diagnosis, and treatment approaches for this challenging bone tumor. The continued exploration of miRNAs in chondrosarcoma holds great promise to improve patient outcomes and improve the lives of individuals affected by this disease.

## Figures and Tables

**Table 1:** This table summarizes miRNA expression, targets, and functional roles in chondrosarcoma, including their levels and place of detection. ↓ and ↑ signifies downregulation and upregulation respectively.

miRNA	Target	Function in CS	Level	Observed in	Link
miR-143-3p/145-5p	FSCN1	Cell adhesion, motility, migration, and cellular interactions	**↓**	Plasma and CS cells	[13]
miR-145, miR-494	SOX9	Inhibit proliferation and invasion	**↓**	Plasma and CS cells	[14,15]
miR-181a	RGS16, CXCR4, VEGF	Enhances VEGF expression, impacts tumor progression	↑	CS cells	[14,16,17,18,19]
miR-26a	PIK3C2alpha/Akt/HIF-alpha pathway	Inhibits angiogenesis by down-regulating VEGF-A	**↓**	CS cells	[14,20]
miR-335	SOX4, TNC	Inhibits metastasis	**↓**	CS cells	[21]
miR-100	mTOR	Suppresses tumor growth and metastasis by inhibiting mTOR, increases cisplatin sensitivity	**↓**	CS cells	[21,22,23]
miR-101	TIMP-3	Promotes cell migration	↑	CS cells	[24]
miR-126, miR-199a, miR-206, miR-452	VEGF-A	Inhibit angiogenesis by down-regulating VEGF-A	**↓**	CS cells	[14,25,26,27,28,29]
miR-125b	ErbB2	Inhibits cell motility and invasiveness, increases doxorubicin sensitivity	**↓**	CS cells	[14]
miR-30a	SOX9, SOX4, RUNX2	Suppresses tumor proliferation, migration, and invasion	**↓**	CS cells	[14,30,31]
miR-138-5p	MACF1	Inhibits osteoblast differentiation	**↓**	CS cells	[21,32,33]
mir-21-5p	CCR7	Suppresses proliferation, migration, and invasion	**↓**	CS cells	[34]
miR-186, miR-27b, miR-624-3p	VEGF-C	Inhibits angiogenesis by down-regulating VEGF-C	**↓**	CS cells	[35,36,37,38]
miR-146a-5p	TXNIP	Inhibits cell proliferation and promotes apoptosis	**↓**	CS cells	[39,40]
miR-491-5p, miR-342-5p	EGFR, Bcl-xL, Bcl-2	Induce apoptosis and autophagy, inhibit EGFR expression	**↓**	CS cells	[41]
miR-454-3p	STAT3, ATG12	Induces apoptosis, inhibits cell growth	**↓**	CS cells	[22]
miR-23b	Src kinase	Inhibits cell proliferation and metastasis	**↓**	CS cells	[22]
miR-497	Cdc25A	Suppresses proliferation and enhances apoptosis	**↓**	CS cells	[21,42]
miR-34	NOTCH1, C-MYC, LMTK3, KLF4	Inhibits cell growth, cell migration and invasion, induces apoptosis	**↓**	CS cells	[43,44]
miR-525	SPON1	Stimulates the production of inflammation factors	**↓**	CS cells	[45]
miR-518b	Rap1b	Promotion of apoptosis and inhibition of cell migration	**↓**	CS cells	[46,47]
miR-631	APLN	Suppresses angiogenesis, metastasis, cell proliferation, increases doxorubicin sensitivity	**↓**	CS cells	[48]

## Data Availability

The authors declare that all data and materials supporting the findings of this study are available in this article. Data supporting the findings of this study are available from the corresponding author upon reasonable request.

## References

[1] Kawamata T., Tomari Y. (2010). Making RISC. Trends Biochem. Sci..

[2] Jo M.H., Shin S., Jung S.R., Kim E., Song J.J., Hohng S. (2015). Human Argonaute 2 Has Diverse Reaction Pathways on Target RNAs. Mol. Cell.

[3] Vasudevan S., Steitz J.A. (2007). AU-rich-element-mediated upregulation of translation by FXR1 and Argonaute 2. Cell.

[4] Truesdell S.S., Mortensen R.D., Seo M., Schroeder J.C., Lee J.H., LeTonqueze O., Vasudevan S. (2012). MicroRNA-mediated mRNA translation activation in quiescent cells and oocytes involves recruitment of a nuclear microRNP. Sci. Rep..

[5] Bukhari S.I.A., Truesdell S.S., Lee S., Kollu S., Classon A., Boukhali M., Jain E., Mortensen R.D., Yanagiya A., Sadreyev R.I. (2016). A Specialized Mechanism of Translation Mediated by FXR1a-Associated MicroRNP in Cellular Quiescence. Mol. Cell.

[6] Orom U.A., Nielsen F.C., Lund A.H. (2008). MicroRNA-10a binds the 5’UTR of ribosomal protein mRNAs and enhances their translation. Mol. Cell.

[7] de Rie D., Abugessaisa I., Alam T., Arner E., Arner P., Ashoor H., Astrom G., Babina M., Bertin N., Burroughs A.M. (2017). An integrated expression atlas of miRNAs and their promoters in human and mouse. Nat. Biotechnol..

[8] Kim Y.K., Kim V.N. (2007). Processing of intronic microRNAs. EMBO J..

[9] Kozomara A., Griffiths-Jones S. (2014). miRBase: Annotating high confidence microRNAs using deep sequencing data. Nucleic Acids Res..

[10] Friedman R.C., Farh K.K., Burge C.B., Bartel D.P. (2009). Most mammalian mRNAs are conserved targets of microRNAs. Genome Res..

[11] Lu J., Getz G., Miska E.A., Alvarez-Saavedra E., Lamb J., Peck D., Sweet-Cordero A., Ebert B.L., Mak R.H., Ferrando A.A. (2005). MicroRNA expression profiles classify human cancers. Nature.

[12] Valihrach L., Androvic P., Kubista M. (2020). Circulating miRNA analysis for cancer diagnostics and therapy. Mol. Asp. Med..

[13] Urdinez J., Boro A., Mazumdar A., Arlt M.J., Muff R., Botter S.M., Bode-Lesniewska B., Fuchs B., Snedeker J.G., Gvozdenovic A. (2020). The miR-143/145 Cluster, a Novel Diagnostic Biomarker in Chondrosarcoma, Acts as a Tumor Suppressor and Directly Inhibits Fascin-1. J. Bone Miner. Res..

[14] Palmini G., Marini F., Brandi M.L. (2017). What Is New in the miRNA World Regarding Osteosarcoma and Chondrosarcoma?. Molecules.

[15] Li J., Wang L., Liu Z., Zu C., Xing F., Yang P., Yang Y., Dang X., Wang K. (2015). MicroRNA-494 inhibits cell proliferation and invasion of chondrosarcoma cells in vivo and in vitro by directly targeting SOX9. Oncotarget.

[16] Sun X., Charbonneau C., Wei L., Chen Q., Terek R.M. (2015). miR-181a Targets RGS16 to Promote Chondrosarcoma Growth, Angiogenesis, and Metastasis. Mol. Cancer Res..

[17] Sun X., Chen Y., Yu H., Machan J.T., Alladin A., Ramirez J., Taliano R., Hart J., Chen Q., Terek R.M. (2019). Anti-miRNA Oligonucleotide Therapy for Chondrosarcoma. Mol. Cancer Ther..

[18] Mutlu S., Mutlu H., Kirkbes S., Eroglu S., Kabukcuoglu Y.S., Kabukcuoglu F., Duymus T.M., Isik M., Ulasli M. (2015). The expression of miR-181a-5p and miR-371b-5p in chondrosarcoma. Eur. Rev. Med. Pharmacol. Sci..

[19] Sun X., Wei L., Chen Q., Terek R.M. (2015). MicroRNA regulates vascular endothelial growth factor expression in chondrosarcoma cells. Clin. Orthop. Relat. Res..

[20] Chai Z.T., Kong J., Zhu X.D., Zhang Y.Y., Lu L., Zhou J.M., Wang L.R., Zhang K.Z., Zhang Q.B., Ao J.Y. (2013). MicroRNA-26a inhibits angiogenesis by down-regulating VEGFA through the PIK3C2alpha/Akt/HIF-1alpha pathway in hepatocellular carcinoma. PLoS ONE.

[21] Yoshitaka T., Kawai A., Miyaki S., Numoto K., Kikuta K., Ozaki T., Lotz M., Asahara H. (2013). Analysis of microRNAs expressions in chondrosarcoma. J. Orthop. Res..

[22] Jeong W., Kim H.J. (2018). Biomarkers of chondrosarcoma. J. Clin. Pathol..

[23] Chang L., Shrestha S., LaChaud G., Scott M.A., James A.W. (2015). Review of microRNA in osteosarcoma and chondrosarcoma. Med. Oncol..

[24] Tsai C.H., Yang D.Y., Lin C.Y., Chen T.M., Tang C.H., Huang Y.L. (2017). Sphingosine-1-phosphate suppresses chondrosarcoma metastasis by upregulation of tissue inhibitor of metalloproteinase 3 through suppressing miR-101 expression. Mol. Oncol..

[25] Liu B., Peng X.C., Zheng X.L., Wang J., Qin Y.W. (2009). MiR-126 restoration down-regulate VEGF and inhibit the growth of lung cancer cell lines in vitro and in vivo. Lung Cancer.

[26] Zhu Q.D., Zhou Q.Q., Dong L., Huang Z., Wu F., Deng X. (2018). MiR-199a-5p Inhibits the Growth and Metastasis of Colorectal Cancer Cells by Targeting ROCK1. Technol. Cancer Res. Treat..

[27] Liu G.T., Huang Y.L., Tzeng H.E., Tsai C.H., Wang S.W., Tang C.H. (2015). CCL5 promotes vascular endothelial growth factor expression and induces angiogenesis by down-regulating miR-199a in human chondrosarcoma cells. Cancer Lett..

[28] Wang C.Q., Huang Y.W., Wang S.W., Huang Y.L., Tsai C.H., Zhao Y.M., Huang B.F., Xu G.H., Fong Y.C., Tang C.H. (2017). Amphiregulin enhances VEGF-A production in human chondrosarcoma cells and promotes angiogenesis by inhibiting miR-206 via FAK/c-Src/PKCdelta pathway. Cancer Lett..

[29] Lin C.Y., Tzeng H.E., Li T.M., Chen H.T., Lee Y., Yang Y.C., Wang S.W., Yang W.H., Tang C.H. (2017). WISP-3 inhibition of miR-452 promotes VEGF-A expression in chondrosarcoma cells and induces endothelial progenitor cells angiogenesis. Oncotarget.

[30] Lu N., Lin T., Wang L., Qi M., Liu Z., Dong H., Zhang X., Zhai C., Wang Y., Liu L. (2015). Association of SOX4 regulated by tumor suppressor miR-30a with poor prognosis in low-grade chondrosarcoma. Tumour Biol..

[31] Jiang D., Zheng X., Shan W., Shan Y. (2016). The overexpression of miR-30a affects cell proliferation of chondrosarcoma via targeting Runx2. Tumour Biol..

[32] Chen Z., Zhao F., Liang C., Hu L., Li D., Zhang Y., Yin C., Chen L., Wang L., Lin X. (2020). Silencing of miR-138-5p sensitizes bone anabolic action to mechanical stimuli. Theranostics.

[33] Zhang L., Yang M., Mayer T., Johnstone B., Les C., Frisch N., Parsons T., Mi Q.S., Gibson G. (2017). Use of MicroRNA biomarkers to distinguish enchondroma from low-grade chondrosarcoma. Connect. Tissue Res..

[34] Li G., Yang Y., Xu S., He M., Zhang Z. (2021). mir-21-5p inhibits the progression of human chondrosarcoma by regulating CCR7/STAT3/NF-kappaB pathway. Connect. Tissue Res..

[35] Su C.M., Tang C.H., Chi M.J., Lin C.Y., Fong Y.C., Liu Y.C., Chen W.C., Wang S.W. (2018). Resistin facilitates VEGF-C-associated lymphangiogenesis by inhibiting miR-186 in human chondrosarcoma cells. Biochem. Pharmacol..

[36] Huang C.Y., Chang A.C., Chen H.T., Wang S.W., Lo Y.S., Tang C.H. (2016). Adiponectin promotes VEGF-C-dependent lymphangiogenesis by inhibiting miR-27b through a CaMKII/AMPK/p38 signaling pathway in human chondrosarcoma cells. Clin. Sci..

[37] Yang W.H., Chang A.C., Wang S.W., Wang S.J., Chang Y.S., Chang T.M., Hsu S.K., Fong Y.C., Tang C.H. (2016). Leptin promotes VEGF-C production and induces lymphangiogenesis by suppressing miR-27b in human chondrosarcoma cells. Sci. Rep..

[38] Lin C.Y., Wang S.W., Chen Y.L., Chou W.Y., Lin T.Y., Chen W.C., Yang C.Y., Liu S.C., Hsieh C.C., Fong Y.C. (2017). Brain-derived neurotrophic factor promotes VEGF-C-dependent lymphangiogenesis by suppressing miR-624-3p in human chondrosarcoma cells. Cell Death Dis..

[39] Parafioriti A., Cifola I., Gissi C., Pinatel E., Vilardo L., Armiraglio E., Di Bernardo A., Daolio P.A., Felsani A., D’Agnano I. (2020). Expression profiling of microRNAs and isomiRs in conventional central chondrosarcoma. Cell Death Discov..

[40] Zhao G., Gu W. (2020). Effects of miR-146a-5p on chondrocyte interleukin-1beta-induced inflammation and apoptosis involving thioredoxin interacting protein regulation. J. Int. Med. Res..

[41] Veys C., Benmoussa A., Contentin R., Duchemin A., Brotin E., Lafont J.E., Saintigny Y., Poulain L., Denoyelle C., Demoor M. (2021). Tumor Suppressive Role of miR-342-5p in Human Chondrosarcoma Cells and 3D Organoids. Int. J. Mol. Sci..

[42] Lu Y., Li F., Xu T., Sun J. (2016). miRNA-497 Negatively Regulates the Growth and Motility of Chondrosarcoma Cells by Targeting Cdc25A. Oncol. Res..

[43] Zajac A., Krol S.K., Rutkowski P., Czarnecka A.M. (2021). Biological Heterogeneity of Chondrosarcoma: From (Epi) Genetics through Stemness and Deregulated Signaling to Immunophenotype. Cancers.

[44] Vares G., Ahire V., Sunada S., Ho Kim E., Sai S., Chevalier F., Romeo P.H., Yamamoto T., Nakajima T., Saintigny Y. (2020). A multimodal treatment of carbon ions irradiation, miRNA-34 and mTOR inhibitor specifically control high-grade chondrosarcoma cancer stem cells. Radiother. Oncol..

[45] Liu B., Song X., Yan Z., Yang H., Shi Y., Wu J. (2019). MicroRNA-525 enhances chondrosarcoma malignancy by targeting F-spondin 1. Oncol. Lett..

[46] Zhang M., Zhou S., Zhang L., Zhang J., Cai H., Zhu J., Huang C., Wang J. (2012). miR-518b is down-regulated, and involved in cell proliferation and invasion by targeting Rap1b in esophageal squamous cell carcinoma. FEBS Lett..

[47] Liang W., Li X., Li Y., Li C., Gao B., Gan H., Li S., Shen J., Kang J., Ding S. (2014). Gallic acid induces apoptosis and inhibits cell migration by upregulating miR-518b in SW1353 human chondrosarcoma cells. Int. J. Oncol..

[48] Chen J.C., Shih H.C., Lin C.Y., Guo J.H., Huang C., Huang H.C., Chong Z.Y., Tang C.H. (2023). MicroRNA-631 Resensitizes Doxorubicin-Resistant Chondrosarcoma Cells by Targeting Apelin. Int. J. Mol. Sci..

[49] Bao X., Ren T., Huang Y., Sun K., Wang S., Liu K., Zheng B., Guo W. (2017). Knockdown of long non-coding RNA HOTAIR increases miR-454-3p by targeting Stat3 and Atg12 to inhibit chondrosarcoma growth. Cell Death Dis..

[50] Tavazoie S.F., Alarcon C., Oskarsson T., Padua D., Wang Q., Bos P.D., Gerald W.L., Massague J. (2008). Endogenous human microRNAs that suppress breast cancer metastasis. Nature.

[51] Huang K., Chen J., Yang M.S., Tang Y.J., Pan F. (2017). Inhibition of Src by microRNA-23b increases the cisplatin sensitivity of chondrosarcoma cells. Cancer Biomark..

[52] Tang X.Y., Zheng W., Ding M., Guo K.J., Yuan F., Feng H., Deng B., Sun W., Hou Y., Gao L. (2016). miR-125b acts as a tumor suppressor in chondrosarcoma cells by the sensitization to doxorubicin through direct targeting the ErbB2-regulated glucose metabolism. Drug Des. Devel Ther..

[53] Chen S.S., Tang C.H., Chie M.J., Tsai C.H., Fong Y.C., Lu Y.C., Chen W.C., Lai C.T., Wei C.Y., Tai H.C. (2019). Resistin facilitates VEGF-A-dependent angiogenesis by inhibiting miR-16-5p in human chondrosarcoma cells. Cell Death Dis..

[54] Tsai C.H., Tsai H.C., Huang H.N., Hung C.H., Hsu C.J., Fong Y.C., Hsu H.C., Huang Y.L., Tang C.H. (2015). Resistin promotes tumor metastasis by down-regulation of miR-519d through the AMPK/p38 signaling pathway in human chondrosarcoma cells. Oncotarget.

[55] Zhu Z., Wang C.P., Zhang Y.F., Nie L. (2014). MicroRNA-100 resensitizes resistant chondrosarcoma cells to cisplatin through direct targeting of mTOR. Asian Pac. J. Cancer Prev..

[56] Mak I.W., Singh S., Turcotte R., Ghert M. (2015). The epigenetic regulation of SOX9 by miR-145 in human chondrosarcoma. J. Cell Biochem..

[57] Liu R.X., Tang W., Zheng B.Y., Yang Y., Li Z.Y., Gui T., Zhang H.T., Liu N., Zha Z.G., Li J.X. (2022). YAP/miR-524-5p axis negatively regulates TXNIP expression to promote chondrosarcoma cell growth. Biochem. Biophys. Res. Commun..

[58] Nicolle R., Ayadi M., Gomez-Brouchet A., Armenoult L., Banneau G., Elarouci N., Tallegas M., Decouvelaere A.V., Aubert S., Redini F. (2019). Integrated molecular characterization of chondrosarcoma reveals critical determinants of disease progression. Nat. Commun..

[59] Tuddenham L., Wheeler G., Ntounia-Fousara S., Waters J., Hajihosseini M.K., Clark I., Dalmay T. (2006). The cartilage specific microRNA-140 targets histone deacetylase 4 in mouse cells. FEBS Lett..

[60] Zhang X., Wang C., Huang C., Yang J., Wang J. (2023). Doxorubicin resistance in breast cancer xenografts and cell lines can be counterweighted by microRNA-140-3p, through PD-L1 suppression. Histol. Histopathol..

[61] Zhang W., Hsu P., Zhong B., Guo S., Zhang C., Wang Y., Luo C., Zhan Y., Zhang C. (2018). MiR-34a Enhances Chondrocyte Apoptosis, Senescence and Facilitates Development of Osteoarthritis by Targeting DLL1 and Regulating PI3K/AKT Pathway. Cell Physiol. Biochem..

[62] Imani S., Zhang X., Hosseinifard H., Fu S., Fu J. (2017). The diagnostic role of microRNA-34a in breast cancer: A systematic review and meta-analysis. Oncotarget.

[63] Wang Z., Ting Z., Li Y., Chen G., Lu Y., Hao X. (2013). microRNA-199a is able to reverse cisplatin resistance in human ovarian cancer cells through the inhibition of mammalian target of rapamycin. Oncol. Lett..

[64] Xu N., Zhang J., Shen C., Luo Y., Xia L., Xue F., Xia Q. (2012). Cisplatin-induced downregulation of miR-199a-5p increases drug resistance by activating autophagy in HCC cell. Biochem. Biophys. Res. Commun..

[65] Li S., Wu Y., Zhang J., Sun H., Wang X. (2020). Role of miRNA-424 in Cancers. Onco Targets Ther..

[66] Vishnubalaji R., Shaath H., Elango R., Alajez N.M. (2020). Noncoding RNAs as potential mediators of resistance to cancer immunotherapy. Semin. Cancer Biol..

[67] Pala D., Kapoor M., Woods A., Kennedy L., Liu S., Chen S., Bursell L., Lyons K.M., Carter D.E., Beier F. (2008). Focal adhesion kinase/Src suppresses early chondrogenesis: Central role of CCN2. J. Biol. Chem..

[68] Li J., Wang Y., Song Y., Fu Z., Yu W. (2014). miR-27a regulates cisplatin resistance and metastasis by targeting RKIP in human lung adenocarcinoma cells. Mol. Cancer.

[69] Qu J., Zhao L., Zhang P., Wang J., Xu N., Mi W., Jiang X., Zhang C., Qu J. (2015). MicroRNA-195 chemosensitizes colon cancer cells to the chemotherapeutic drug doxorubicin by targeting the first binding site of BCL2L2 mRNA. J. Cell Physiol..

[70] Gu Y.L., Rong X.X., Wen L.T., Zhu G.X., Qian M.Q. (2017). miR-195 inhibits the proliferation and migration of chondrocytes by targeting GIT1. Mol. Med. Rep..

[71] Xiao J., Chen X., Xu L., Zhang Y., Yin Q., Wang F. (2014). PDGF regulates chondrocyte proliferation through activation of the GIT1- and PLCgamma1-mediated ERK1/2 signaling pathway. Mol. Med. Rep..

[72] Dai H., Xu L.Y., Qian Q., Zhu Q.W., Chen W.X. (2019). MicroRNA-222 promotes drug resistance to doxorubicin in breast cancer via regulation of miR-222/bim pathway. Biosci. Rep..

[73] Zeng L.P., Hu Z.M., Li K., Xia K. (2016). miR-222 attenuates cisplatin-induced cell death by targeting the PPP2R2A/Akt/mTOR Axis in bladder cancer cells. J. Cell Mol. Med..

[74] Yoshizuka M., Nakasa T., Kawanishi Y., Hachisuka S., Furuta T., Miyaki S., Adachi N., Ochi M. (2016). Inhibition of microRNA-222 expression accelerates bone healing with enhancement of osteogenesis, chondrogenesis, and angiogenesis in a rat refractory fracture model. J. Orthop. Sci..

[75] Jamialahmadi K., Zahedipour F., Karimi G. (2021). The role of microRNAs on doxorubicin drug resistance in breast cancer. J. Pharm. Pharmacol..

[76] Jiang P., Jia W., Wei X., Zhang X., Wang C., Li B., Song T., Yang J., Zhu D., Meng Y. (2017). MicroRNA-146a regulates cisplatin-resistance of non-small cell lung cancer cells by targeting NF-kappaB pathway. Int. J. Clin. Exp. Pathol..

[77] Mao G., Zhang Z., Huang Z., Chen W., Huang G., Meng F., Zhang Z., Kang Y. (2017). MicroRNA-92a-3p regulates the expression of cartilage-specific genes by directly targeting histone deacetylase 2 in chondrogenesis and degradation. Osteoarthr. Cartil..

[78] Yamashita S., Miyaki S., Kato Y., Yokoyama S., Sato T., Barrionuevo F., Akiyama H., Scherer G., Takada S., Asahara H. (2012). L-Sox5 and Sox6 proteins enhance chondrogenic miR-140 microRNA expression by strengthening dimeric Sox9 activity. J. Biol. Chem..

[79] Galoian K.A., Guettouche T., Issac B., Qureshi A., Temple H.T. (2014). Regulation of onco and tumor suppressor MiRNAs by mTORC1 inhibitor PRP-1 in human chondrosarcoma. Tumour Biol..

[80] Jie J., Liu D., Wang Y., Wu Q., Wu T., Fang R. (2022). Generation of MiRNA sponge constructs targeting multiple MiRNAs. J. Clin. Lab. Anal..

[81] Xie B., Zhao Z., Liu Q., Wang X., Ma Z., Li H. (2019). CircRNA has_circ_0078710 acts as the sponge of microRNA-31 involved in hepatocellular carcinoma progression. Gene.

[82] Zhu Q., Lu G., Luo Z., Gui F., Wu J., Zhang D., Ni Y. (2018). CircRNA circ_0067934 promotes tumor growth and metastasis in hepatocellular carcinoma through regulation of miR-1324/FZD5/Wnt/beta-catenin axis. Biochem. Biophys. Res. Commun..

[83] Graham L.D., Pedersen S.K., Brown G.S., Ho T., Kassir Z., Moynihan A.T., Vizgoft E.K., Dunne R., Pimlott L., Young G.P. (2011). Colorectal Neoplasia Differentially Expressed (CRNDE), a Novel Gene with Elevated Expression in Colorectal Adenomas and Adenocarcinomas. Genes. Cancer.

[84] Huan J., Xing L., Lin Q., Xui H., Qin X. (2017). Long noncoding RNA CRNDE activates Wnt/beta-catenin signaling pathway through acting as a molecular sponge of microRNA-136 in human breast cancer. Am. J. Transl. Res..

[85] Lee S., Choi E.J., Jin C., Kim D.H. (2005). Activation of PI3K/Akt pathway by PTEN reduction and PIK3CA mRNA amplification contributes to cisplatin resistance in an ovarian cancer cell line. Gynecol. Oncol..

[86] Chang S., Chen B., Wang X., Wu K., Sun Y. (2017). Long non-coding RNA XIST regulates PTEN expression by sponging miR-181a and promotes hepatocellular carcinoma progression. BMC Cancer.

[87] Hu B., Cai H., Zheng R., Yang S., Zhou Z., Tu J. (2017). Long non-coding RNA 657 suppresses hepatocellular carcinoma cell growth by acting as a molecular sponge of miR-106a-5p to regulate PTEN expression. Int. J. Biochem. Cell Biol..

[88] Zhuang L.K., Yang Y.T., Ma X., Han B., Wang Z.S., Zhao Q.Y., Wu L.Q., Qu Z.Q. (2016). MicroRNA-92b promotes hepatocellular carcinoma progression by targeting Smad7 and is mediated by long non-coding RNA XIST. Cell Death Dis..

[89] Hoffman Y., Bublik D.R., Ugalde A.P., Elkon R., Biniashvili T., Agami R., Oren M., Pilpel Y. (2016). 3’UTR Shortening Potentiates MicroRNA-Based Repression of Pro-differentiation Genes in Proliferating Human Cells. PLoS Genet..

[90] Cheng C., Bhardwaj N., Gerstein M. (2009). The relationship between the evolution of microRNA targets and the length of their UTRs. BMC Genom..

